# Instrumental musical self-concept in secondary school: the influence of socio-demographic and educational variables in non-specialised educational environments

**DOI:** 10.3389/fpsyg.2026.1886757

**Published:** 2026-08-12

**Authors:** María Begoña Zarza-Alzugaray, Oscar Casanova, Francisco Javier Zarza-Alzugaray

**Affiliations:** 1 Department of Educational Sciences, Faculty of Humanities and Education, University of La Rioja, Logroño, Spain; 2 Musical, Plastic and Body Expression Department, Faculty of Education, University of Zaragoza, Zaragoza, Spain

**Keywords:** educational context, music as a school subject, music instrument practice, musical self-concept, secondary education, socio-educational variables

## Abstract

Musical self-concept is a relevant construct in education research, due to its relationship with motivational, academic, and contextual variables. Our study aimed to analyse the levels of musical self-concept among adolescents in secondary education and to examine the relationship between musical self-concept and several sociodemographic and educational variables within the framework of school music taught as a subject in Spain. Our sample consisted of 980 students ages 12–18, enrolled in secondary schools in the autonomous regions of Aragon and Navarra. For data collection, we used the “instrumental musical self-concept” scale, along with a further questionnaire designed to collect contextual variables. We conducted variance analyses and a multiple linear regression analysis using the stepwise method. Our results show several significant differences in the level of an individual’s instrumental musical self-concept by sex, habitat (urban/rural), whether they practice an instrument, and whether they receive educational support. We found that general instrumental practice is the main predictor of instrumental musical self-concept, both inside and outside the classroom. These findings contribute to a refined understanding of musical self-concept as a construct primarily driven by sustained instrumental practice and shaped by contextual educational conditions rather than solely by static socio-demographic factors. Our results highlight the need to consider several socio-educational and contextual variables when designing secondary school programmes and activities.

## 1 Introduction

Education research has shown that the construct of musical self-concept is formed in function of educational, social, and family contexts (Clark and Lonsdale, 2023; Luo and Guan, 2025; Petersen et al., 2023; Ruismäki and Tereska, 2006). Recent cross-cultural research has further suggested that musical self-concept is shaped not only by individual musical experiences, but also by broader social comparison processes and culturally mediated perceptions of musical competence (Petersen et al., 2023; Arens et al., 2025).

Recent investigations have examined musical self-concept in adolescents in educational settings; most have done so in specifically musical contexts. Zubeldia et al. (2018) analysed the roles of age and gender variables in conservatory students’ musical self-concept. They found differences in means by gender depending on the type of musical activity; however, among adolescents, the age variable yielded no significant differences in any of the dimensions they studied. Subsequently, Blanco et al. (2019) examined the relationship between musical self-concept and the motivational climate perceived by students enrolled in secondary-level music schools (conservatorios profesionales). According to their results, male students scored significantly higher on the factor of self-perceived musical competency. They also found negative correlations between the age variable and the dimension of musical competency. More recently, Lohbeck (2023) and Arens et al. (2025) extended the generalized internal/external frame of reference model to the music domain, showing that musical self-concept is strongly influenced by social and dimensional comparison processes within educational settings.

Further studies have associated musical self-context with other contextual variables: academic performance (Granada et al., 2012), motivation (Blanco, 2022; Blanco et al., 2019), attributional styles (Zubeldia et al., 2018), and emotional intelligence (Olarte et al., 2022). Recent evidence has also linked musical self-concept to wellbeing-related variables such as stress regulation, flourishing, and mastery pleasure in music learning contexts (Kovács et al., 2025; Yu and Xu, 2025).

Certain studies, without investigating musical self-concept per se, have shown that practising a musical instrument can be beneficial to self-concept in general (Degé et al., 2014; Retamero, 2021). This, in turn, positively impacts adolescents’ academic performance (Degé et al., 2014; Guhn et al., 2020; Schmidt, 2005), motivation, and causal attributions (Evans and Liu, 2019; McPherson et al., 2015; Schatt, 2023; Schmidt, 2005).

As educational research on the construct of musical self-concept progresses, it has become apparent that musical self-concept needs to be analysed by taking into account several different musical activities (Goñi et al., 2020; Palacios et al., 2009; Zubeldia et al., 2017), including instrumental practice. Current theoretical approaches also emphasize the multidimensional and context-dependent nature of musical self-concept, suggesting that different musical activities may generate distinct perceptions of competence and self-evaluation (Petersen et al., 2023). Therefore, based on Vispoel’s (2003) definition of self-concept and Ruismäki and Tereska's (2006) definition of musical self-concept, we propose that instrumental musical self-concept is an individual’s perception of their musical competence and performance as someone who plays a musical instrument.

In Spain, however, we found no studies that analysed musical self-concept in the context of general education. The current study aimed to compare levels of instrumental musical self-concept depending on several socio-demographic and educational variables, and to analyse the relationships between those variables and instrumental musical self-concept.

Taking the current theories of self-concept and musical self-concept into account, we proposed two hypotheses:

H1: that we would find significant differences in levels of instrumental musical self-concept among groups delimited and defined by several socio-educational variables.

H2: that we would find significant relationships among the following variables: instrumental musical self-concept, sex, academic year, educational support, rural vs. urban habitat, number of hours per week devoted to playing a musical instrument during music class hours at school, practising a musical instrument outside school hours, and extracurricular musical training.

## 2 Materials and methods

### 2.1 Participants

The sample for this study consisted of 980 students enrolled in compulsory secondary education (ESO): 463 boys (47.2%) and 517 girls (52.8%), aged between 12 and 18 (*X̄* = 13,887, SD = 1.3425), attending schools in the Spanish Autonomous Communities of Aragon (93.06%, *n* = 912) and Navarre (6.93%, *n* = 68).

A non-probability convenience sampling strategy was employed, based on the accessibility of participating schools. Schools were selected according to their willingness to participate in the study and the inclusion of music as part of the compulsory secondary education curriculum.

As a selection criterion, we included only students who were taking music as a subject in secondary school at the time they completed the questionnaire. In accordance with the recommendations of Buendía et al. (1998) regarding indispensable cohort characteristics for research, we did not include students with cognitive disabilities.

In terms of academic level, 39.4% of these students were in their first year of ESO, i.e., obligatory secondary education (*n* = 386), 1.1% of them were in their second year (*n* = 11), 50.7% of them were in third year (*n* = 497), and 8.8% were in fourth (*n* = 86).

### 2.2 Variables and measurement tools

For the specific purposes of this study, we developed an *ad hoc* questionnaire to collect socio-demographic variables (sex, academic year, rural vs. urban municipality) and educational variables (participation in educational support programmes, number of weekly hours devoted to playing a musical instrument during music lessons, instrument practice outside school hours, and extracurricular musical training). As the questionnaire collected objective contextual information rather than latent psychological constructs, no psychometric validation procedure was required. Nevertheless, the items were reviewed by the research team to ensure their clarity, relevance, and appropriateness. To measure instrumental musical self-concept, we used the instrumental musical self-concept scale (Zarza, 2024), consisting of 16 items on a 7-point Likert-type scale ranging from 1 = totally disagree to 7 = totally agree. This scale measures students’ self-perception of instrumental practice across four dimensions: music instrument competency (Items 1, 14, 16, 23, and 25), music instrument incompetence (Items 3, 8, 20, 24, and 26), social motivation and personal development (Items 6, 13, 19, and 21), and emotional factors (Items 5 and 9).

The scale was developed through a multi-stage validation process. The initial phase involved the development and refinement of the initial item pool. Subsequently, the preliminary version was evaluated by expert judges to establish content validity, followed by data collection and exploratory factor analysis, which led to the removal of items with inadequate psychometric performance. The final version was validated using a larger, independent sample through descriptive item analysis, exploratory factor analysis based on the maximum likelihood extraction method, parallel analysis, and confirmatory factor analysis. A detailed description of the development and psychometric validation of the instrumental musical self-concept scale is provided in Zarza (2024). The confirmatory factor analysis supported the proposed four-factor structure and showed satisfactory model fit (*χ^2^* = 147.226, df = 97, *χ^2^*/df = 1.518, CFI = 0.979, RMSEA = 0.032), providing evidence of construct validity. In the present study, the scale showed high internal consistency (Cronbach’s *α* = 0.840).

### 2.3 Procedure and analysis

To collect the data, we contacted the schools’ principals and music subject teachers by email, explained our research, and asked them if they would be willing to participate voluntarily. Once they had given their consent, informed consent forms were distributed to parents and students, along with an accompanying letter explaining our research. Once the required permissions had been obtained, the questionnaires were administered to the students by the school management or music teachers. The questionnaires were completed during regular school hours in the classroom under the supervision of the school management or the music teacher, who was available to clarify procedural questions when necessary but did not influence students’ responses. The questionnaires were administered online, anonymously and voluntarily via Google Docs. The response time to complete all the questionnaires ranged from 20 to 30 min. Prior to the data collection process, we requested and obtained corresponding approval and authorisation from the Research Ethics Committee of the Autonomous Community of Aragon (CEICA).

To analyse the behavior of a dependent (criterion) variable based on a constant (fixed) factor, we performed ANOVA-based mean-comparison analyses. In our research design, we considered instrumental musical self-concept as the dependent variable, and the following factors as independent variables: sex, academic year, educational support, rural vs. urban habitat, hours per week devoted to music instrument practice as part of the school music subject, instrument practice outside school hours, and extracurricular musical training outside school hours.

To examine the joint contribution of the independent variables (sex, educational support, rural vs. urban habitat, weekly hours devoted to playing an instrument during music lessons, instrument practice outside school hours, and extracurricular musical training), a forward stepwise multiple linear regression analysis was performed. The independent variables were entered sequentially according to their statistical contribution to the dependent variable. This procedure was employed to identify the variables that made the greatest independent contribution to instrumental musical self-concept while retaining only those predictors that significantly improved model fit (Tourón et al., 2023). Although stepwise procedures have recognised limitations, including the risk of overfitting and data-driven variable selection, they remain appropriate when the aim is to identify the variables with the greatest explanatory contribution among a set of potential predictors.

Data analysis was conducted according to the recommendations of Tourón et al. (2023). We used the SPSS statistical package in version 25.0.

## 3 Results

In the first section, we present the results obtained from one-way ANOVA analysis. It was performed from a bivariate perspective, analysing differences between two independent groups based on a single factor from the variance.

Based on those one-way ANOVA results, the second section presents result from multiple linear regression.

### 3.1 Variance analysis (ANOVA)

In the following subsections, to ease comprehension, only significant differences among variables are featured. To obtain a better grasp of the meaning of “incompetence,” we only studied direct scores for that factor.

#### 3.1.1 Sex

ANOVA variance analysis showed that the following factors had significantly different means in groups according to sex (male or female): music instrument competency (*F* = 9.072, *ρ* = 0.003; *Levene* = 1.138, *ρ* = 0.286), music instrument incompetence (*F* = 6.516, *ρ* = 0.011; *Levene* = 0.498, *ρ* = 0.480), social motivation and personal development (*F* = 36.134, *ρ* = 0.000; *Levene* = 0.723, *ρ* = 0.395), emotional factor (*F* = 28.527, *ρ* = 0.000; *Levene* = 0.206, *ρ* = 0.650), and scores on the “music instrument self-concept” scale (*F* = 31.761, *ρ* = 0.000; *Levene* = 0.169, *ρ* = 0.681). Females achieved significantly higher means on each of these factors, except incompetence, where they felt less incompetent (Table 1).

**Table 1:** ANOVA of instrumental musical self-concept by sex (M/F).

Factor	Sex	*N*	Mean	DT	*F*	Sig.	Effect size (*η*^2^)
Competence	Male	463	22.897	6.687	9.072	0.003	0.009
	Female	517	24.152	6.687			
	Total	980	23.559	6.542			
Incompetence	Male	463	16.130	6.394	6.516	0.011	0.007
	Female	517	15.108	6.130			
	Total	980	15.591	6.274			
Social motivation and personal development	Male	463	14.318	5.838	36.134	0.000	0.036
	Female	517	16.586	5.946			
	Total	980	15.514	6.000			
Emotion	Male	463	8.271	3.509	28.527	0.000	0.028
	Female	517	9.459	3.441			
	Total	980	8.898	3.522			
“Instrumental musical self-concept” scale	Male	463	69.356	16.112	31.761	0.000	0.031
	Female	517	75.089	15.701			
	Total	980	72.380	16.144			

#### 3.1.2 Educational support programmes (yes/no)

Comparing between students who were enrolled in educational support programmes and those who were not, the Mann–Whitney *U* test showed significant differences among means in the following factors: music instrument competency (Z* = −2.713, *ρ* = 0.007), music instrument incompetence (Z* = −3.231, *ρ* = 0.001), and instrumental musical self-concept (Z* = −2.859, *ρ* = 0.004). Students who were not following educational support programmes displayed significantly higher means in the factor of music instrument competence and instrumental musical self-concept, and lower scores on the incompetence score, i.e., they felt less incompetent (Table 2).

**Table 2:** Mann–Whitney *U* test of instrumental musical self-concept according to educational support programmes.

Factor	Ed. support	*N*	Mean	DT	*Z* ^*^	Sig.
Competency	No	948	23.661	6.537	−2.713	0.007
	Yes	32	20.531	6.048		
	Total	980	23.559	6.542		
Incompetence	No	948	15.470	6.251	−3.231	0.001
	Yes	32	19.187	5.980		
	Total	980	15.591	6.274		
“Instrumental musical self-concept” scale	No	948	72.640	16.154	−2.859	0.004
	Yes	32	64.687	14.005		
	Total	980	72.380	16.144		

^*^Mann–Whitney *U* test.

#### 3.1.3 Rural vs. urban

Comparing subjects who lived in an urban municipality to those who live in a rural municipality, ANOVA variance analysis showed significant differences among means in the following two factors: social motivation and personal development (*F* = 8.617, *ρ* = 0.003; *Levene* = 1.641, *ρ* = 0.200) on the one hand, and scores on the “instrumental musical self-concept” scale (*F* = 6.679, *ρ* = 0.010; *Levene* = 5.458, *ρ* = 0.020; *Brown-Forsythe* = 6.285, *ρ* = 0.012) on the other. Students from rural areas scored higher on both factors (Table 3).

**Table 3:** ANOVA of instrumental musical self-concept according to urban vs. rural.

Factor	Municipality	*N*	Mean	DT	*F*	Sig.	Effect size (*η*^2^)
Social motivation and personal development	Urban	650	15.115	5.895	8.617	0.003	0.009
	Rural	330	16.301	6.136			
	Total	980	15.514	6.000			
“Instrumental musical self-concept” scale	Urban	650	71.433	15.570	6.679	0.010	0.007
	Rural	330	74.245	17.092			
	Total	980	72.380	16.144			

#### 3.1.4 Practising a musical instrument outside school schedule hours

Comparing students who practiced a musical instrument outside school schedule hours with those who did not, ANOVA variance analysis showed that the following factors yielded significant differences among means between the two groups defined by that independent variable: competency (*F* = 200.367, *ρ* = 0.000; *Levene* = 4.732, *ρ* = 0.030; *Brown-Forsythe* = 226.805, *ρ* = 0.000), incompetence (*F* = 55.980, *ρ* = 0.000; *Levene* = 0.002, *ρ* = 0.962), social motivation and personal development (*F* = 270.490, *ρ* = 0.000; *Levene* = 9.797, *ρ* = 0.002; *Brown-Forsythe* = 315.327, *ρ* = 0.000), emotional factor (*F* = 46.824, *ρ* = 0.000; *Levene* = 5.319, *ρ* = 0.021; *Brown-Forsythe* = 52.276, *ρ* = 0.000), and scores on the “instrumental musical self-concept” scale (*F* = 288.325, *ρ* = 0.000; *Levene* = 0.023, *ρ* = 0.880). Students who played an instrument outside school schedule hours scored significantly higher on each of those factors except instrumental musical incompetence (Table 4).

**Table 4:** ANOVA of instrumental musical self-concept according to whether the student plays an instrument outside of school or not.

Factor	Plays instr. outside school	*N*	Mean	DT	*F*	Sig.	Effect size (*η*^2^)
Musical competency	No	753	22.079	6.111	200.367	0.000	0.170
	Yes	227	28.470	5.443			
	Total	980	23.559	6.542			
Incompetence	No	753	16.392	6.123	55.980	0.000	0.054
	Yes	227	12.933	6.044			
	Total	980	15.591	6.274			
Social motivation and personal development	No	753	13.982	5.473	270.490	0.000	0.217
	Yes	227	20.599	4.742			
	Total	980	15.514	06.000			
Emotion	No	753	8.485	3.518	46.824	0.000	0.046
	Yes	227	10.268	3.175			
	Total	980	22.257	19.795			
“Instrumental musical self-concept” scale	No	753	68.153	14.230	288.325	0.000	0.228
	Yes	227	86.404	14.078			
	Total	980	72.380	16.144			

#### 3.1.5 Extracurricular musical training

Comparing students who received extracurricular musical training (private, music school) with those who did not, ANOVA variance analysis showed that the following factors yielded significant differences among means between the two groups defined by that independent variable: musical competency (*F* = 198.504, *ρ* = 0.000; *Levene* = 18.503, *ρ* = 0.000; *Brown-Forsythe* = 282.809, *ρ* = 0.000), incompetence (*F* = 31.806, *ρ* = 0.000; *Levene* = 3.274, *ρ* = 0.071), social motivation and personal development (*F* = 220.008, *ρ* = 0.000; *Levene* = 14.374, *ρ* = 0.000; *Brown-Forsythe* = 306.701, *ρ* = 0.000), emotional factor (*F* = 46.930, *ρ* = 0.000; *Levene* = 3.072, *ρ* = 0.080) and scores on the “instrumental musical self-concept” scale (*F* = 236.642, *ρ* = 0.000; *Levene* = 0.535, *ρ* = 0.465). Students who received musical training outside of school scored higher on each one of those factors, except incompetence (Table 5).

**Table 5:** ANOVA of instrumental musical self-concept according to whether the student receives musical training outside of school or not.

Factor	Musical training (extracurricular)	*N*	Mean	DT	*F*	Sig.	Effect size (*η*^2^)
Musical competency	No	834	22.435	6.153	198.504	0.000	0.169
	Yes	146	29.979	4.769			
	Total	980	23.559	6.542			
Incompetence	No	834	16.057	6.059	31.806	0.000	0.031
	Yes	146	12.931	6.818			
	Total	980	15.591	6.274			
Social motivation and personal development	No	834	14.439	5.583	220.008	0.000	0.184
	Yes	146	21.657	4.398			
	Total	980	15.514	6.00			
Emotion	No	834	8.583	3.489	46.930	0.000	0.046
	Yes	146	10.698	3.156			
	Total	980	8.898	3.522			
“Instrumental musical self-concept” scale	No	834	69.400	14.664	236.642	0.000	0.195
	Yes	146	89.404	13.477			
	Total	980	72.380	16.144			

#### 3.1.6 Hours dedicated to playing an instrument during music class in school

Comparing means according to the amount of hours dedicated to playing an instrument during music class in school, ANOVA variance analysis showed significant differences between the two groups defined by that independent variable in the following factors: musical competency (*F* = 31.800, *ρ* = 0.000; *Levene* = 2.926, *ρ* = 0.087), social motivation and personal development (*F* = 39.241, *ρ* = 0.000; *Levene* = 0.342, *ρ* = 0.559), emotional factor (*F* = 13.666, *ρ* = 0.000; *Levene* = 1.180, *ρ* = 0.278), and the “Musical Instrumental Self-Concept” scale (*F* = 33.410, *ρ* = 0.000; *Levene* = 0.961, *ρ* = 0.327). Students who played an instrument for more hours during music class scored higher on each one of these factors (Table 6).

**Table 6:** ANOVA of instrumental musical self-concept according to the number of hours devoted to playing an instrument during music class at school.

Factor	Playing instrument	*N*	Mean	DT	*F*	Sig.	Effect size (*η*^2^)
Musical competency	0–1 h	645	22.723	6.594	31.800	0.000	0.031
	2–3 h	335	25.169	6.137			
	Total	980	23.559	6.542			
Social motivation and personal development	0–1 h	645	14.665	5.917	39.241	0.000	0.039
	2–3 h	335	17.149	5.827			
	Total	980	15.514	6.000			
Emotion	0–1 h	645	8.600	3.561	13.666	0.000	0.014
	2–3 h	335	9.471	3.378			
	Total	980	8.898	3.522			
“Instrumental musical self-concept” scale	0–1 h	645	70.267	15.706	33.410	0.000	0.033
	2–3 h	335	76.450	16.220			
	Total	980	72.380	16.144			

Overall, the reported effect sizes indicate that the socio-demographic variables (sex and municipality) were associated with small effects on instrumental musical self-concept (*η^2^* = 0.007–0.036). By contrast, the educational variables directly related to instrumental practice showed the largest effect sizes. Instrument practice outside school hours yielded large effects (*η^2^* = 0.170–0.228), while extracurricular musical training also showed large effects (*η^2^* = 0.169–0.195) for musical competence, social motivation and personal development, and the overall instrumental musical self-concept score.

### 3.2 Regression

Given the significant differences observed between instrumental musical self-concept and the socio-demographic and educational variables, we conducted a forward stepwise multiple regression analysis to identify the variables that made the greatest contribution to instrumental musical self-concept. We also considered it necessary to examine these variables simultaneously in order to explain instrumental musical self-concept from a broader perspective.

**Figure 1:**
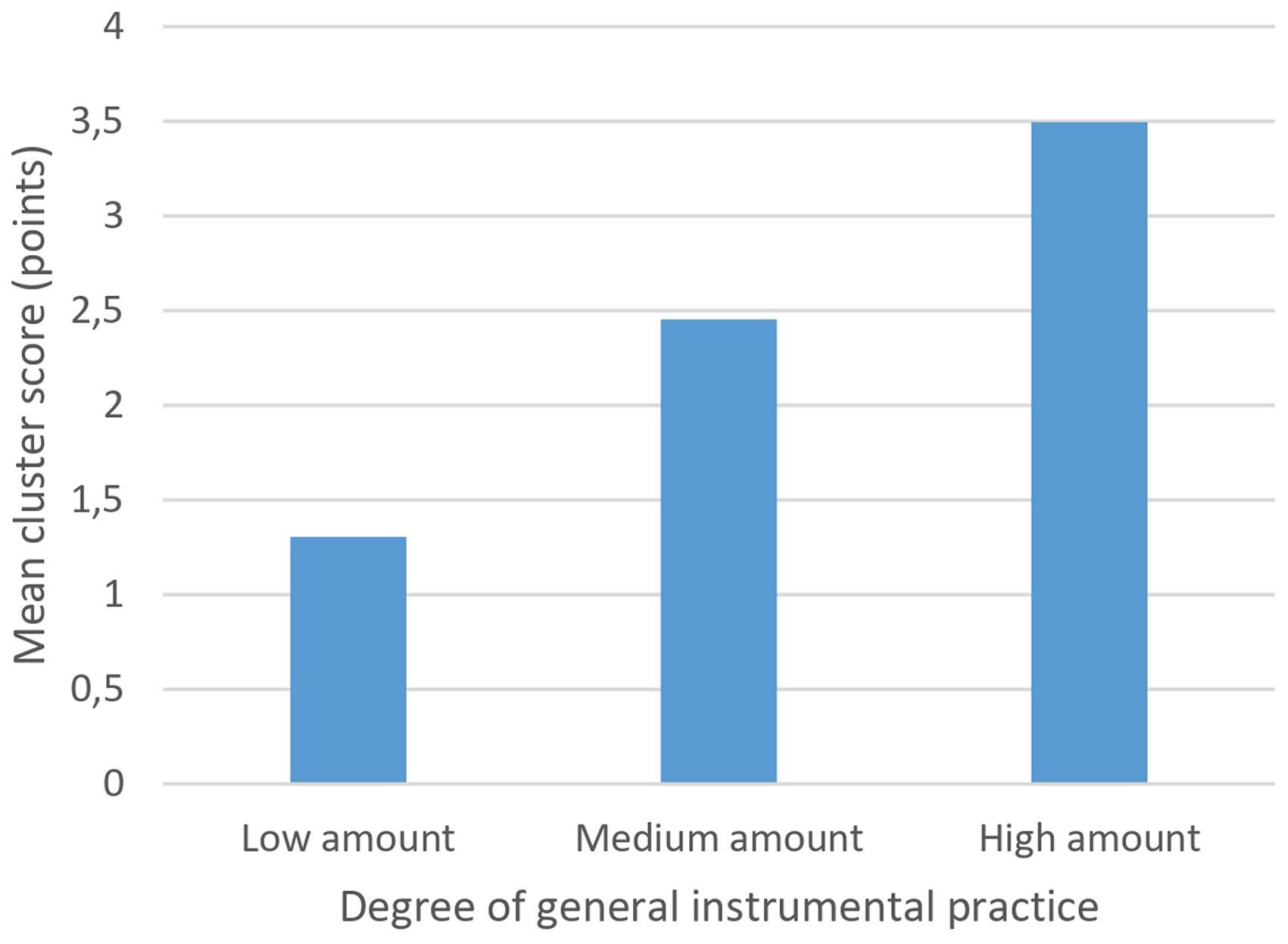
Clusters of degree of general instrumental practice.

To summarise the information provided by the pedagogical variables related to instrumental practice, a *k*-means cluster analysis was performed to identify distinct profiles of students’ instrumental engagement. Specifically, the clustering procedure included three variables: instrument practice outside school hours, extracurricular musical training, and hours devoted to instrumental practice during music lessons. This approach was preferred over analysing each variable separately because it enabled the construction of broader participation profiles, facilitating a more parsimonious interpretation of students’ patterns of instrumental engagement while reducing redundancy among closely related variables and simplifying the interpretation of the subsequent regression model. The number of clusters was set at three (*k* = 3) to obtain an interpretable classification of students according to their level of instrumental engagement (low, medium, and high). The *k*-means analysis identified three profiles of instrumental participation. As shown in Figure 1, these profiles corresponded to students with low (*X̄* = 1.304, *n* = 746), medium (*X̄* = 2.454, *n* = 99), and high (*X̄*= 3.496, *n* = 135) levels of instrumental engagement. These groups should be interpreted as exploratory analytical profiles rather than as fixed categories of students. As *k*-means is an exploratory partitioning technique, no additional cluster validation indices were calculated. Instead, the adequacy of the selected solution was supported by the clear interpretability of the resulting profiles and by the statistically significant differences observed across the variables included in the clustering procedure (*F* = 1374.285, *p* = 0.000).

Table 7 displays the final model for this approximation (*F* = 91.416, *ρ* = 0.000; *DW* = 1.835), which explains 27.0% of the variance. Furthermore, the residuals are independent, as the Durbin-Watson statistic lies between 1.5 and 2.5. Regression diagnostics indicated that the assumptions of the linear regression model were satisfactorily met. Inspection of the histogram and normal probability plot of the standardised residuals showed that the residuals were approximately normally distributed. Likewise, visual inspection of the scatterplot of standardised residuals against standardised predicted values revealed no evidence of heteroscedasticity. Regarding multicollinearity diagnostics, Table 7 reports the collinearity statistics (Tolerance and VIF values) for the variables retained in the final regression model, whereas Table 8 presents the corresponding statistics for the excluded variables. No evidence of multicollinearity was observed among the predictors. Tolerance values ranged from 0.987 to 1.000, corresponding to VIF values close to 1 and well below the commonly accepted threshold of 5, indicating that the regression coefficients were not affected by multicollinearity.

**Table 7:** Instrumental musical self-concept: linear regression model.

Model	Predictor	Standardized coefficients	*t*	Sig.	*F*	Corrected *R*-squared	Collinearity statistics	
							VIF	Minimum tolerance
1	Sex	0.177	5.636	0.000	31.761	0.030	1.000	1.000
2	Sex	0.176	5.594	0.000	19.546	0.037	1.000	1.000
	Ed. support	−0.084	−2.670	0.008			1.000	1.000
3	Sex	0.175	5.578	0.000	15.249	0.042	0.999	1.001
	Ed. support	−0.083	−2.654	0.080			0.999	1.001
	Rural vs. urban	0.079	2.537	0.011			1.000	1.000
4	Sex	0.134	4.876	0.000	91.416	0.270	0.992	1.008
	Ed. support	−0.057	−2.081	0.038			0.996	1.004
	Rural vs. urban	0.056	2.041	0.042			0.997	1.003
	Cluster General instrumental practice	0.481	17.483	0.000			0.987	1.013

**Table 8:** Variables excluded from the linear regression model.

Predictor		Beta	*t*	Sig.	Partial correlation	Collinearity statistics		
						Tolerance	VIF	Minimum tolerance
1	Ed. support	-.084	−2.670	0.008	−0.085	1.000	1.000	1.000
	Rural vs. urban	.080	2.554	0.011	0.081	1.000	1.000	1.000
	Cluster General instrumental practice	.487	17.687	0.000	0.492	0.992	1.008	0.992
2	Rural vs. urban	.079	2.537	0.011	0.081	1.000	1.000	0.999
	Cluster General instrumental practice	.483	17.576	0.000	0.490	0.989	1.011	0.989
3	Cluster General instrumental practice	.481	17.483	0.000	0.489	0.987	1.013	0.987

The first model included only the sex variable, which exerted a significant influence (*t* = 5.636, *ρ* = 0.000; *β* = 0.177; *Corrected R-squared* = 0.030; *F* = 31.761, *ρ* = 0.000).

The second model, apart from the sex variable (*t* = 5.594; *ρ* = 0.000; *β* = 0.176), also included educational support, which exerted a significant influence (*t* = −2.670, *ρ* = 0.008; *β* = −0.084; *corrected R2* = 0.037; *F* = 19.546, *ρ* = 0.000).

In the third model, the sex variable continued to exert a significant influence (*t* = 5.578, *p* = 0.000; *β* = 0.175), along with the rural/urban variable (*t* = 2.537, *ρ* = 0.011; *β* = 0.079). However, in this model, educational support no longer had a significant influence (*t* = −2.654, *ρ* = 0.080; *β* = −0.083; *corrected R2* = 0.042; *F* = 15.249, *ρ* = 0.000).

In the fourth and final model, the variables of sex (*t* = 4.876, *ρ* = 0.000; *β* = 0.134), rural/urban habitat (*t* = 2.041, *ρ* = 0.042; *β* = 0.056), and general instrumental practice (*t* = 17.483, *ρ* = 0.000; *β* = 0.481) continued to exert a significant influence. Moreover, the variable of educational support reappeared and exerted a significant influence (*t* = −2.081, *ρ* = 0.038; *β* = −0.057; *corrected R2* = 0.270; *F* = 91.416, *ρ* = 0.000).

## 4 Discussion and conclusions

In this study, we aimed to analyse levels of instrumental musical self-concept by sex, academic year, educational support, rural/urban habitat, and instrumental practice, and to examine the interrelationships among these variables.

Female respondents achieved higher mean scores on all factors, except for incompetence (i.e., they perceived themselves as being less incompetent).

These results are at odds with certain previous studies of music school students, including Blanco (2022), which found that competency was the only factor to show significant differences, with male students scoring higher. Another previous study with results that differed from ours was that conducted by Zubeldia et al. (2018), who observed significant differences between males and females according to the type of musical activity. These discrepancies may reflect the multidimensional and context-dependent nature of musical self-concept recently highlighted in the literature, according to which self-perceptions in music may vary depending on the educational context, the type of musical activity, and the social comparison processes involved (Arens et al., 2025; Lohbeck, 2023).

Academic year did not play a statistically significant role in any of the factors; this observation is consistent with the results of Zubeldia et al. (2018), who found no significant differences between the pre-adolescent and mid-adolescent age groups.

Students who were not following an educational support programme had a more positive instrumental musical self-concept and saw themselves as more competent and less incompetent. These results are in line with previous studies that have associated musical self-concept with academic performance (Granada et al., 2012), motivation (Blanco, 2022; Blanco et al., 2019), attributional styles (Zubeldia et al., 2018), and emotional intelligence (Olarte et al., 2022). Recent studies have also linked musical self-concept to mastery motivation, mastery pleasure, and wellbeing-related variables, suggesting that students with more positive perceptions of their musical competence tend to show greater motivational engagement and psychological flourishing (Janurik et al., 2023; Kovács et al., 2025; Yu and Xu, 2025).

Educational support programmes are specifically designed to help students with academic or motivational difficulties; this explains why those respondents had a lower musical self-concept level (according to current musical self-concept theory). It would also explain the relationship among several variables in these respondents’ instrumental musical self-concept.

Moreover, our results showed that students living in rural areas achieved higher scores on the “Social Motivation and Personal Development” factor and the “instrumental musical self-concept” scale.

Although these latter results cannot be compared with previous studies, they do highlight the relevance of habitat (rural vs. urban) in the configuration of a subject’s instrumental musical self-concept. These results underscore the importance of a subject’s social environment in shaping their self-concept (Marsh et al., 2014), thereby suggesting a new line of research. In this regard, recent international studies have emphasized the role of family, school, and sociocultural environments in the development of musical self-concept and musical participation (Clark and Lonsdale, 2023; Luo and Guan, 2025).

Students who practised an instrument outside school obtained significantly higher scores on all factors, except incompetence, where the reverse was true. Moreover, students who played an instrument for more hours during the week in music class in school achieved higher scores in music competency, social motivation, personal development, the emotional factor, and instrumental musical self-concept. These results are in line with the theory of Ruismäki and Tereska (2006), who posited that a greater amount of training in music, particularly on an instrument, increases instrumental musical self-concept. Similarly, recent studies have shown that greater involvement in musical learning activities is associated with higher levels of mastery motivation, musical competence, and self-perceived musical ability (Janurik et al., 2023; Kovács et al., 2025).

Our regression analysis confirmed our second hypothesis (H2), based on previous research, that a significant relationship exists among the variables of instrumental musical self-concept, sex, educational support, rural/urban habitat, and instrument practice.

Our results likewise show that the student’s sex (M/F) plays a significant role in shaping their musical self-concept (in our case, instrumental musical self-concept). This finding aligns with the studies by Zubeldia et al. (2018) and Blanco (2022). It should be taken into account in school music curricula and lesson plans.

Our results regarding the educational support variable also confirm previous research demonstrating a significant relationship between musical self-concept and academic performance (Granada et al., 2012), attributional styles (Zubeldia et al., 2018), and motivation (Blanco, 2022). They also seem to agree with previous studies on general and academic self-concept, which reported similar relationships (Chávez-Becerra et al., 2020; Martín, 2011; Martín-Romero and Sánchez-López, 2021; Ramos-Díaz et al., 2017). Educational practice thus needs to take students’ psychological and contextual characteristics into account.

Our results regarding the rural vs. urban habitat factor could not be compared with any previous self-concept studies—either general or specifically musical—conducted in Spain, as the habitat factor has not yet been taken into account in this area of research. Notwithstanding, our results support the theory that self-concept is shaped by an individual’s social environment (Marsh et al., 2014). The present study thus opens new lines of research on the social environment’s influence on the construction of musical self-concept, considering differences between rural and urban habitats. These findings are also consistent with recent approaches that conceptualize musical self-concept as a socially and culturally mediated construct rather than as a purely individual characteristic (Arens et al., 2025; Petersen et al., 2023).

Concerning instrumental practice, our results coincide with previous studies showing how musical self-concept (Ruismäki and Tereska, 2006) or, where applicable, academic self-concept (Degé et al., 2014; Guhn et al., 2020; Retamero, 2021) is influenced by instrumental practice, whether in music class or as an extracurricular activity or training. These results highlight the importance of instrumental training for students, both inside and outside the secondary school classroom, in shaping their self-concept. Furthermore, recent evidence suggests that continued participation in musical activities may reinforce positive self-perceptions through motivational, emotional, and social reinforcement processes (Janurik et al., 2023; Yu and Xu, 2025).

Beyond statistical significance, the observed effect sizes indicate that instrumental musical self-concept is influenced more strongly by students’ engagement in instrumental practice than by their socio-demographic characteristics. From an educational perspective, this finding reinforces previous evidence suggesting that sustained instrumental practice contributes to the development of musical competence, motivation, and positive musical self-perceptions (Janurik et al., 2023; Kovács et al., 2025; Ruismäki and Tereska, 2006).

This study has some limitations that should be taken into account. First, its cross-sectional design did not allow us to establish causal relationships among the variables we were analysing. Second, the use of self-reports may lead to biases stemming from students’ subjective perceptions. Furthermore, given that the sample was limited to the two Spanish autonomous communities of Aragon and Navarre, these results cannot be generalised. Future research could adopt longitudinal designs, allowing researchers to observe the gradual evolution of instrumental musical self-concept during the secondary education stage. It would also be relevant to achieve greater precision on socioeconomic, family, and geographical variables. Similarly, we suggest extending this research to other communities and educational contexts, combining quantitative and qualitative methodologies to further explore the construction of instrumental musical self-concept. Regarding the relationship between rural/urban habitat and instrumental musical self-concept, we suggest that future research should include additional variables that may also influence its construction. Future studies could also examine the influence of social comparison processes, cultural context, and wellbeing-related variables on the development of instrumental musical self-concept across adolescence (Arens et al., 2025; Lohbeck, 2023; Yu and Xu, 2025).

In conclusion, the findings suggest that promoting regular instrumental practice both within and beyond the secondary school music classroom may contribute to strengthening students’ instrumental musical self-concept. Schools should therefore consider expanding opportunities for instrumental learning through classroom activities, extracurricular programmes, and collaborations with local music schools or community music initiatives. In addition, teachers should be aware that contextual factors, such as students’ educational support needs and learning environments, may influence the development of instrumental musical self-concept and should therefore be considered when designing inclusive music education practices. Finally, the findings reinforce current theoretical perspectives that conceptualise musical self-concept as a multidimensional construct shaped by motivational, contextual, and sociocultural influences throughout students’ educational experiences.

## Data Availability

The raw data supporting the conclusions of this article will be made available by the authors, without undue reservation.
